# Medial temporal tau predicts memory decline in cognitively unimpaired elderly

**DOI:** 10.1093/braincomms/fcac325

**Published:** 2022-12-09

**Authors:** Angela T H Kwan, Saman Arfaie, Joseph Therriault, Zahra Azizi, Firoza Z Lussier, Cecile Tissot, Mira Chamoun, Gleb Bezgin, Stijn Servaes, Jenna Stevenon, Nesrine Rahmouni, Vanessa Pallen, Serge Gauthier, Pedro Rosa-Neto

**Affiliations:** Faculty of Medicine, University of Ottawa, Ottawa, ON K1H 8L1, Canada; Translational Neuroimaging Laboratory, The McGill University Research Centre for Studies in Aging, Douglas Mental Health University Institute, Le Centre Intégré Universitaire de Santé et de Services Sociaux (CIUSSS) de l'Ouest-de-l'Île-de-Montréal, Montreal, QC H4H 1R3, Canada; Translational Neuroimaging Laboratory, The McGill University Research Centre for Studies in Aging, Douglas Mental Health University Institute, Le Centre Intégré Universitaire de Santé et de Services Sociaux (CIUSSS) de l'Ouest-de-l'Île-de-Montréal, Montreal, QC H4H 1R3, Canada; Department of Neurology and Neurosurgery, McGill University, Montreal, QC H3A 1A1, Canada; Department of Medicine, McGill University Health Centre, Montreal, QC H3G 2M1, Canada; Translational Neuroimaging Laboratory, The McGill University Research Centre for Studies in Aging, Douglas Mental Health University Institute, Le Centre Intégré Universitaire de Santé et de Services Sociaux (CIUSSS) de l'Ouest-de-l'Île-de-Montréal, Montreal, QC H4H 1R3, Canada; Department of Neurology and Neurosurgery, McGill University, Montreal, QC H3A 1A1, Canada; Department of Medicine, McGill University Health Centre, Montreal, QC H3G 2M1, Canada; Translational Neuroimaging Laboratory, The McGill University Research Centre for Studies in Aging, Douglas Mental Health University Institute, Le Centre Intégré Universitaire de Santé et de Services Sociaux (CIUSSS) de l'Ouest-de-l'Île-de-Montréal, Montreal, QC H4H 1R3, Canada; Department of Neurology and Neurosurgery, McGill University, Montreal, QC H3A 1A1, Canada; Translational Neuroimaging Laboratory, The McGill University Research Centre for Studies in Aging, Douglas Mental Health University Institute, Le Centre Intégré Universitaire de Santé et de Services Sociaux (CIUSSS) de l'Ouest-de-l'Île-de-Montréal, Montreal, QC H4H 1R3, Canada; Department of Neurology and Neurosurgery, McGill University, Montreal, QC H3A 1A1, Canada; Translational Neuroimaging Laboratory, The McGill University Research Centre for Studies in Aging, Douglas Mental Health University Institute, Le Centre Intégré Universitaire de Santé et de Services Sociaux (CIUSSS) de l'Ouest-de-l'Île-de-Montréal, Montreal, QC H4H 1R3, Canada; Department of Neurology and Neurosurgery, McGill University, Montreal, QC H3A 1A1, Canada; Translational Neuroimaging Laboratory, The McGill University Research Centre for Studies in Aging, Douglas Mental Health University Institute, Le Centre Intégré Universitaire de Santé et de Services Sociaux (CIUSSS) de l'Ouest-de-l'Île-de-Montréal, Montreal, QC H4H 1R3, Canada; Department of Neurology and Neurosurgery, McGill University, Montreal, QC H3A 1A1, Canada; Translational Neuroimaging Laboratory, The McGill University Research Centre for Studies in Aging, Douglas Mental Health University Institute, Le Centre Intégré Universitaire de Santé et de Services Sociaux (CIUSSS) de l'Ouest-de-l'Île-de-Montréal, Montreal, QC H4H 1R3, Canada; Department of Neurology and Neurosurgery, McGill University, Montreal, QC H3A 1A1, Canada; Translational Neuroimaging Laboratory, The McGill University Research Centre for Studies in Aging, Douglas Mental Health University Institute, Le Centre Intégré Universitaire de Santé et de Services Sociaux (CIUSSS) de l'Ouest-de-l'Île-de-Montréal, Montreal, QC H4H 1R3, Canada; Department of Neurology and Neurosurgery, McGill University, Montreal, QC H3A 1A1, Canada; Translational Neuroimaging Laboratory, The McGill University Research Centre for Studies in Aging, Douglas Mental Health University Institute, Le Centre Intégré Universitaire de Santé et de Services Sociaux (CIUSSS) de l'Ouest-de-l'Île-de-Montréal, Montreal, QC H4H 1R3, Canada; Department of Neurology and Neurosurgery, McGill University, Montreal, QC H3A 1A1, Canada; Translational Neuroimaging Laboratory, The McGill University Research Centre for Studies in Aging, Douglas Mental Health University Institute, Le Centre Intégré Universitaire de Santé et de Services Sociaux (CIUSSS) de l'Ouest-de-l'Île-de-Montréal, Montreal, QC H4H 1R3, Canada; Department of Neurology and Neurosurgery, McGill University, Montreal, QC H3A 1A1, Canada; Translational Neuroimaging Laboratory, The McGill University Research Centre for Studies in Aging, Douglas Mental Health University Institute, Le Centre Intégré Universitaire de Santé et de Services Sociaux (CIUSSS) de l'Ouest-de-l'Île-de-Montréal, Montreal, QC H4H 1R3, Canada; Department of Neurology and Neurosurgery, McGill University, Montreal, QC H3A 1A1, Canada; Translational Neuroimaging Laboratory, The McGill University Research Centre for Studies in Aging, Douglas Mental Health University Institute, Le Centre Intégré Universitaire de Santé et de Services Sociaux (CIUSSS) de l'Ouest-de-l'Île-de-Montréal, Montreal, QC H4H 1R3, Canada; Department of Neurology and Neurosurgery, McGill University, Montreal, QC H3A 1A1, Canada; Department of Medicine, McGill University Health Centre, Montreal, QC H3G 2M1, Canada

**Keywords:** Alzheimer’s disease, Braak I–II, memory, PET, tau

## Abstract

Alzheimer’s disease can be detected in living people using *in vivo* biomarkers of amyloid-β and tau, even in the absence of cognitive impairment during the preclinical phase. [^18^F]-MK-6420 is a high-affinity PET tracer that quantifies tau neurofibrillary tangles, but its ability to predict cognitive changes associated with early Alzheimer’s disease symptoms, such as memory decline, is unclear. Here, we assess the prognostic accuracy of baseline [^18^F]-MK-6420 tau-PET for predicting longitudinal memory decline in asymptomatic elderly individuals. In a longitudinal observational study, we evaluated a cohort of cognitively normal elderly participants (*n* = 111) from the translational biomarkers in ageing and dementia study (data collected between October 2017 and July 2020, with a follow-up period of 12 months). All participants underwent tau-PET with [^18^F]-MK-6420 and amyloid-β PET with [^18^F]-AZD-4694. The exclusion criteria included the presence of head trauma, stroke or other neurological disorders. There were 111 eligible participants selected based on the availability of amyloid-β PET, tau-PET, MRI and APOEɛ4 genotyping. Among these participants, the mean standard deviation age was 70.1 (8.6) years; 20 (18%) were tau-PET-positive and 71 of 111 (63.9%) were women. A significant association between the baseline Braak Stages I–II [^18^F]-MK-6240 standardized uptake value ratio positivity and change in composite memory score were observed at the 12-month follow-up, after correcting for age, sex and years of education [logical memory and Rey Auditory Verbal Learning Test, standardized beta = −0.52 (−0.82–0.21), *P* < 0.001, for dichotomized tau-PET and −1.22 (−1.84−(−0.61)], *P* < 0.0001, for continuous tau-PET]. Moderate cognitive decline was observed for A + T + over the follow-up period, whereas no significant change was observed for A−T+, A + T- and A-T-, although it should be noted that the A−T + group was small. Our results indicate that baseline tau neurofibrillary tangle pathology is associated with longitudinal changes in memory function, supporting the use of [^18^F]-MK-6420 PET to predict the likelihood of asymptomatic elderly individuals experiencing future memory decline. Overall, [^18^F]-MK-6420 PET is a promising tool for predicting memory decline in older adults without cognitive impairment at baseline. This is of critical relevance as the field is shifting towards a biological model of Alzheimer’s disease defined by the aggregation of pathologic tau. Therefore, early detection of tau pathology using [^18^F]-MK-6420 PET provides us with hope that living patients with Alzheimer’s disease may be diagnosed during the preclinical phase before it is too late.

## Teaser

In a longitudinal observational study, Kwan, Arfaie *et al*. used [^18^F]-MK-6420, a high-affinity tau-PET tracer, to provide evidence that the presence of tau in the medial temporal lobe predicts longitudinal memory decline in cognitively normal elderly individuals during the preclinical phase of Alzheimer’s disease.

## Graphical Abstract

**Graphical abstract:**
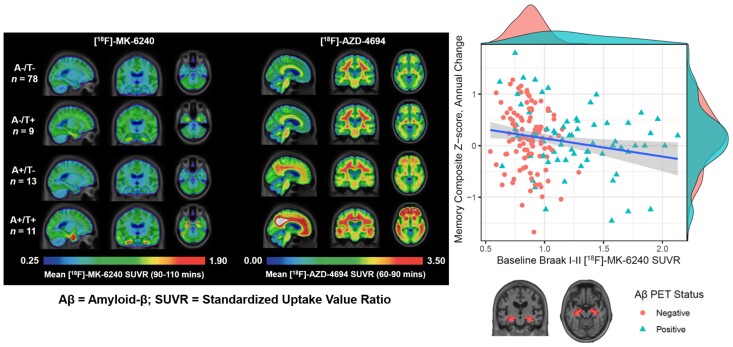

## Introduction

Post-mortem studies show that amyloid-β (Aβ) plaques and tau neurofibrillary tangles, the defining hallmarks of Alzheimer’s disease, are commonly observed in cognitively unimpaired (CU) elderly.^1,2^ This suggests that there is a time window—a preclinical phase—where biological Alzheimer’s disease can be detected in the absence of clinically significant cognitive decline using *in vivo* biomarkers of Aβ and tau.^3,4^ With the recent FDA approval of the first tau tracer, [^18^F]-flortaucipir (AV-1451 or T807), Alzheimer’s disease molecular imaging is approaching clinical use.^5^ Specifically, *in vivo* tau biomarkers derived from PET imaging modalities have shown promise as predictors of future cognitive changes prior to the clinical onset of dementia symptoms.^3,6,7^ The ability to predict memory decline in CU individuals is an important step in defining and detecting biological Alzheimer’s disease *in vivo* and during the preclinical phase.^8^

Previous cross-sectional and longitudinal neuroimaging studies have demonstrated an association between tau pathology and cognitive decline in the CU elderly.^9–11^ However, the [^18^F]-flortaucipir tau-PET tracer used in these studies is prone to age-related off-target binding and its uptake only aligns accurately for Braak Stages IV or higher, suggesting limited sensitivity towards early tau accumulation.^12,13^ The emergence and use of [^18^F]-MK-6420 in clinical studies have shown subnanomolar affinity towards detecting tau neurofibrillary tangles in Alzheimer’s disease and decreased non-specific binding in the brain.^6,14^ This enables greater detection of early tau pathology in CU individuals during the asymptomatic preclinical period, allowing for a better understanding of its association with cognition and other Alzheimer’s disease biomarkers.

In this study, we use the high-affinity tau-PET tracer [^18^F]-MK-6420, focusing on imaging the medial temporal regions to detect very early tau in CU elderly, as opposed to the temporal meta-region of interest for later tau aggregation, at levels observed in Alzheimer’s disease.^10^ Specifically, we examined the prognostic accuracy of baseline [^18^F]-MK-6420 PET for predicting longitudinal memory decline in initially CU elderly, who have been stratified based on the presence of a tau-PET signal in the medial temporal lobe.

## Methods

### Participants

CU participants (*n* = 111), defined by a clinical data repository of zero, were recruited for the translational biomarkers in ageing and dementia cohort as part of the McGill Centre for Studies in Ageing, which investigates biomarker trajectories and interactions as initiators of dementia.^15^ All participants underwent Aβ-PET with [^18^F]-AZD-4694, tau-PET with [^18^F]-MK-6240 and structural MRI. Apolipoprotein E (APOE) genotyping was conducted using the polymerase chain reaction amplification technique. This was followed by restriction enzyme digestion, standard gel resolution and visualization processes. Inclusion criteria included the ability to speak English or French, good overall health, no contraindications to MRI and sufficient visual and auditory ability to comply with a neuropsychological evaluation. The exclusion criteria included the presence of other neurological disorders, stroke or head trauma. This study’s protocol was approved by the Douglas Mental Health University Institute Research Ethics Board and McGill University’s Institutional Review Board. Informed written consent was acquired from all patients.

### PET image acquisition and processing

Each participant underwent a T_1_-weighted MRI, which was utilized for co-registration. PET scans were obtained using the Siemens high-resolution research tomography at the Montreal Neurological Institute. The [^18^F]-MK-6240 images were acquired 90–110 min after injection and scans were reconstructed via the use of the ordered subset expectation maximization algorithm on a 4D volume with four frames (4 × 300 s). The [^18^F]-AZD-4694 images were acquired 40–70 min after injection and scans were reconstructed via use of the ordered subset expectation maximization algorithm on a 4D volume with three frames (3 × 600 s). A 6-min transmission scan was promptly performed following each PET acquisition with a rotating Caesium-137point source to correct for attenuation. Moreover, each image was adjusted for dead time, decay, as well as random and scattered coincidences. T_1_-weighted images were corrected for non-uniformity and field distortion and then processed with an in-house pipeline. Subsequently, PET images were automatically registered to the T_1_-weighted image space and the T_1_-weighted images were linearly and nonlinearly registered to the Alzheimer’s disease neuroimaging initiative template space. To reduce the risk of meningeal spillover within neighbouring brain regions, [^18^F]-MK-6240 images were meninges-striped in native space prior to their transformations and blurring.^16^ Next, a PET non-linear registration was carried out with linear and non-linear transformations from the T_1_-weighted image to the Alzheimer’s disease neuroimaging initiative space and the PET to T_1_-weighted image registration with advanced normalization tools. PET images were spatially smoothed to obtain a final resolution of 8 mm full width at half maximum. The [^18^F]-MK-6240 standardized uptake value ratio (SUVR) and [^18^F]-AZD-4694 SUVR maps were developed by setting the reference region as the inferior cerebellar grey matter and cerebellar grey matter, respectively. A global estimated [^18^F]-AZD-4694 SUVR value was obtained for each participant by averaging SUVR from the orbitofrontal, parietal, precuneus, prefrontal, temporal, anterior and posterior cingulate cortices.^17^

### Neuropsychological assessment

All participants in this study underwent the following clinical assessments: clinical dementia rating, cerebrovascular disease risk via the Hachinski ischaemic scale and mini-mental state examination. Neuropsychological memory exams were carried out by trained neuropsychologists. Memory scores were evaluated with the Rey auditory verbal learning test (RAVLT) and a logical memory test.^15^

### MRI acquisition and processing

Structural MRI data were obtained on a 3 T Siemens Magnetom using a standard head coil for all individuals at the Montreal Neurologic Institute. A fluid-attenuated inversion recovery sequence was also included in the MRI protocol, which was utilized to quantify white matter hyperintensity (WMH) volume. Freesurfer (v6.0) was used to quantify cortical thickness measurements. The Alzheimer’s disease signature meta-region of interest was computed according to the weighted average of the bilateral entorhinal, inferior temporal, middle temporal and fusiform cortices. The WMH volume was determined via the lesion prediction algorithm as established in the LST toolbox version 2.0.15 in scientific principles of medicine, calculating a probability of WMH per voxel. A visual examination was performed for all segmented WMH images. Next, a binary WMH image was characterized for WMH probability > 0.5.^15^

### Statistical analysis

Baseline demographic and clinical data were evaluated using *t*-tests and *χ*^2^ tests. Other statistical analyses were conducted via the R statistical software, version 3.5.3 with two-sided statistical significance set at α = 0.05. Descriptive statistics were written as frequency (%) for categorical variables; the mean [standard deviation (SD)] and median were reported for normally distributed numerical variables. For non-normally distributed data, appropriate non-parametric tests were performed. A student's *t*-test and the non-parametric Wilcoxon–Mann–Whitney test were utilized to compare the memory scores between tau-positive and tau-negative individuals. Any missing values in the models were addressed through pairwise deletion (available case analysis). Multivariable linear regression was employed to assess the relationship between the change in logical memory and RAVLT scores and tau level from baseline to 12 months follow-up. A composite standardized measure of memory (logical memory, RAVLT) was obtained by calculating the *Z*-scores of each test and averaging the scores.^18^ Standardized beta coefficients were reported for each model and the models were adjusted for the following covariants: age, sex, years of education and Aβ-PET levels.^18^ Aβ-PET positivity at baseline was determined based on an [^18^F]-AZD-4694 threshold of ≥ 1.55 SUVR.8 tau-PET positivity at baseline was determined based on an [^18^F]-MK-6240 Braak Stages I–II threshold level of ≥ 1.23.^19^

## Results

### Baseline demographic characteristics

Demographic features of the study sample, including age and [^18^F]-AZD-4694 and MRI-based atrophy clinical measures for all participants and by dichotomized tau groups, are presented in Table 1.

**Table 1:** Baseline demographic characteristics of the study sample

Variable: categorical: *n* (%) numerical: mean (SD), median	All participants^a^	Tau−^a^	Tau +^a^	*P*-value^b^
	(*n* = 111)	(*n* = 91)	(*n* = 20)	
Age at MRI	70.1 (8.6), 71.3	69.3 (9.0), 70.2	74.0 (4.6), 73.9	0.01
Sex (female)	71 (63.9%)	55 (60.4%)	16 (80%)	0.1
Years of education	15.5 (3.7), 15	15.7 (3.7), 16	14.9 (3.8), 13.5	0.3
APOEɛ4 carrier	28 (25.2%)	23 (25.3%)	5 (25%)	< 0.001
[^18^F]-AZD-4694-PET-positive	24 (21.6%)	13 (14.3%)	11 (55%)	< 0.001
Mini-Mental State Examination	29.1 (1.1), 29	29.2 (1.1), 30	29 (1.3), 29	0.3
Hippocampal volume (Hippocampal volume), mm^3^	3.5 (0.4), 3.5	3.6 (0.4), 3.6	3.4 (0.4), 3.4	0.1
WMH, mL	6.0 (6.0), 4.2	5.8 (5.7), 3.8	6.6 (7.2), 5.3	0.4

^a^ The results are presented as frequency (%) for categorical variables and mean (SD) and median for numerical variables.

^b^ *P*-values for the differences in baseline between tau-positive and -negative individuals were determined using the Wilcoxon–Mann–Whitney test for numerical variables and the *χ*^2^ test for categorical variables.

**Figure 1:**
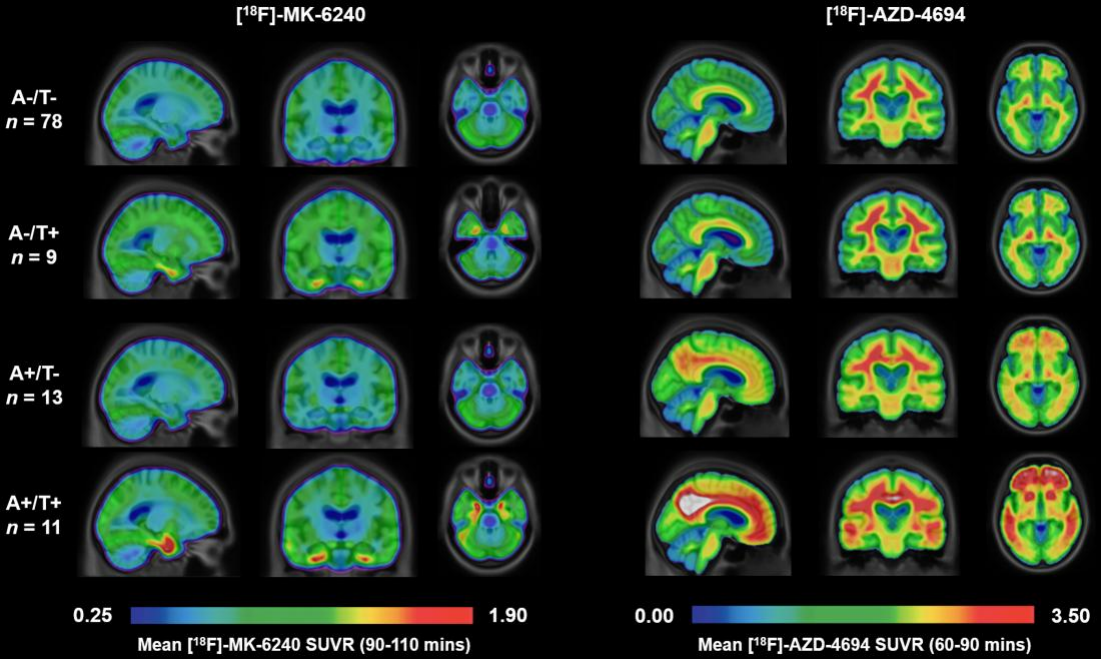
**Parametric [^18^F]-MK-6240 and [^18^F]-AZD-4694 images stratified by biomarker group.** Mean parametric [^18^F]-MK-6240 *(left)* and [^18^F]-AZD-4694 *(right)* SUVR images are shown for each biomarker group (A−T−, A−T+, A + T− and A + T+). Individuals in the A + T + group had the highest [^18^F]-MK-6240 uptake in the medial temporal lobe. Individuals with A + T + showed the largest cortical [^18^F]-AZD-4694 uptake, as compared with the A−T + or A + T− groups.

A total of 111 CU individuals were included in the present study. Participants were from 35 to 86 years of age. The mean (SD) age was 70.1 (8.6) years at the time of their MRI scans; 71 of 111 (63.9%) were women, 20 (18%) were tau-PET-positive, and 24 (21.6%) were Aβ-PET-positive. Group mean parametric images of entorhinal [^18^F]-MK-6420 SUVR and global [^18^F]-AZD-4694 SUVR displaying positivity thresholds are shown in Fig. 1.

The majority of subjects, 70% (*n* = 78), were classified as A−T−, 8% (*n* = 9) as A−T+, 12% (*n* = 13) as A + T− and the remaining 10% (*n* = 11) as A + T + . Within the tau-positive group, 55% of the participants also exhibited Aβ positivity (*n* = 11, *P* < 0.001; Table 1). Individuals who were A−/T + showed elevated [^18^F]-MK-6420 SUVR in the entorhinal cortex, whereas the A+/T + group showed elevated [^18^F]-MK-6420 SUVR extending into the medial temporal lobe and greater neocortex (Fig. 1). The [^18^F]-AZD-4694 SUVR was lower for the A+/T− group throughout the cortex and subcortex than individuals who were A+/T + (Fig. 1).

### Biomarkers and longitudinal prospective cognition

**Figure 2:**
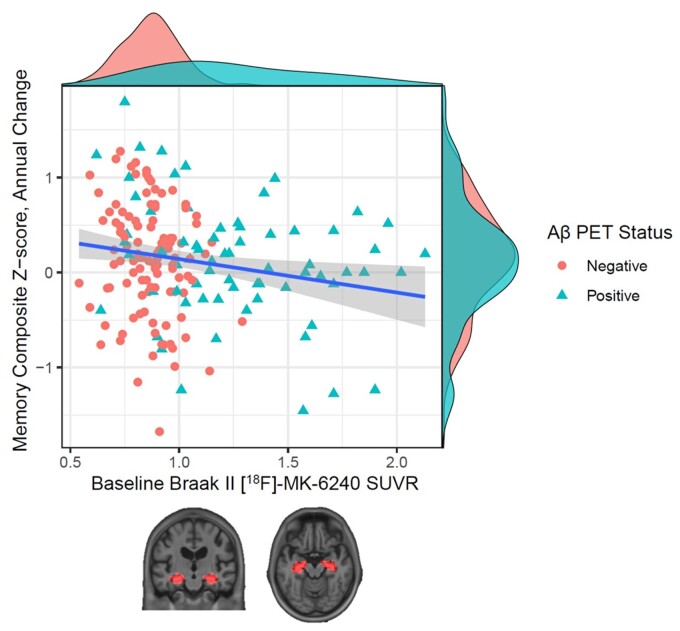
**Association between baseline Braak Stages I–II [^18^F]-MK-6240 SUVR and memory decline, stratified by neocortical [^18^F]-AZD-4694 SUVR.** Density plots illustrated on the *x* and *y* axes display the distribution of data for baseline Braak Stage II SUVR and memory composite *Z*-scores, respectively. In cognitively unimpaired participants, [^18^F]-MK-6240 SUVR levels in Braak Stages I and II regions were associated with annual changes in the memory composite *Z*-score [continuous SUVR measures beta estimate: −1.22, 95% CI: (−1.84−−0.61)), *P* < 0.0001]. Below the density plot, the brain regions where tau-PET was sampled are the hippocampus and the entorhinal cortex.

Prospective cognitive trajectories were examined by plotting annual memory composite *Z*-score changes versus baseline Braak Stages I–II [^18^F]-MK-6240 SUVR stratified by neocortical [^18^F]-AZD-4694 SUVR (Fig. 2).

Multivariable linear regression analyses examining main effects of [^18^F]-MK-6420 PET status in Braak Stages I–II regions on longitudinal memory decline indicated a significant association at 12 months follow-up for dichotomized and continuous SUVR measures {beta estimate: −0.52, 95% confidence interval (CI): (−0.82 to 0.21), *P* < 0.001; Table 2 (**A**) and beta estimate: −1.22, 95% CI: [−1.84−(−0.61)], *P* < 0.0001; Table 2 (**B**), respectively}.

**Table 2:** Main effects of [^18^F]-MK-6420 and [^18^F]-AZD-4694 PET status on longitudinal memory decline in cognitively unimpaired participants. Tau-PET and Aβ-PET positivity were dichotomized in (A), as compared with assessment on a continuous SUVR scale in (B). The analyses performed in (C) and (D) were based on dichotomized Aβ status, for Aβ positive and Aβ negative individuals, respectively

(A)	Medial temporal tau-PET		
	Beta (95% CI)	*t*-value	*P*-value
**Braak Stage II [^18^F]-MK-6240-PET positivity**	−0.52 (−0.82–0.21)	−3.36	0.001
**[^18^F]-AZD-4694-PET Positivity**	0.24 (−0.05–0.53)	1.67	0.1
**Age**	0.008 (−0.01–0.03)	1.67	0.38
**Sex (Male)**	−0.06 (−0.28–0.17)	−0.51	0.61
**Years of education**	−0.01 (−0.03–0.05)	−0.46	0.67
	**Medial Temporal tau-PET**		
**(B)**	**Beta (95% CI)**	***t*-value**	***P*-value**
**Medial temporal [^18^F]-MK-6240-SUVR**	−1.22 (−1.84−(−0.61))	−3.96	0.0001
**Neocortical [^18^F]-AZD-4694-SUVR**	0.27 (−0.007–0.56)	1.93	0.056
**Age**	0.003 (−0.015–0.022)	0.39	0.70
**Sex (male)**	−0.006 (−0.23–0.21)	−0.06	0.95
**Years of education**	−0.01 (−0.03–0.05)	−0.46	0.67
	**Medial Temporal Tau-PET**		
**(C)**	**Beta (95% CI)**	***t*-value**	***P*-value**
**Braak Stage II [^18^F]-MK-6240-PET positivity**	−1.22 (−2.19–0.26)	−2.64	0.015
**Age at MRI**	−0.0006 (−0.053–0.052)	−0.02	0.98
**Sex (male)**	−0.21 (−0.74–0.32)	−0.82	0.42
**Years of education**	0.03 (−0.07–0.21)	0.35	0.72
	**Medial Temporal Tau-PET**		
**(D)**	**Beta (95% CI)**	***t*-value**	***P*-value**
**Braak Stage II [^18^F]-MK-6240-PET positivity**	−0.318 (−0.73–0.09)	−1.54	0.127
**Age at MRI**	−0.0046 (−0.015–0.024)	0.45	0.65
**Sex (male)**	0.07 (−0.18–0.32)	−0.56	0.57
**Years of education**	−0.01 (−0.01–0.04)	−0.51	0.62

Multivariable linear regression revealed a significant change in logical memory and RAVLT annual scores for individuals that were A + T + [beta estimate: −1.22, 95% CI: −2.19–0.26, *P* < 0.015; Table 2 (**C**)], but an insignificant change for A-T + [beta estimate: −0.318, 95% CI: −0.73–0.09, *P* < 0.127; Table 2 (**D**)]. The main effects of [^18^F]-AZD-4694 PET status on longitudinal memory decline were not statistically significant at 12 months follow-up for dichotomized and continuous SUVR measures [beta estimate: 0.24, 95% CI: (−0.05–0.53), *P* < 0.1; Table 2 (**A**) and beta estimate: 0.27, 95% CI: (−0.007–0.56), *P* = 0.056; Table 2  **(B**), respectively].

### PET biomarkers and age

**Figure 3:**
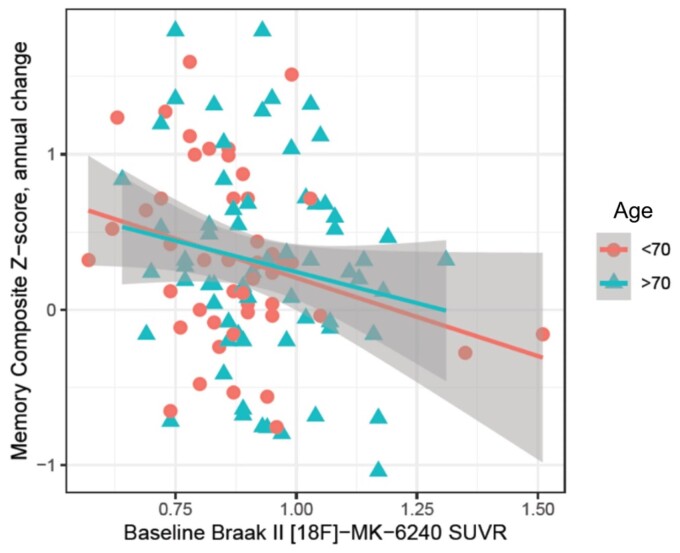
**Association between baseline Braak Stages I–II [^18^F]-MK-6240 SUVR and longitudinal memory decline, stratified by age.** In cognitively unimpaired participants, no statistically significant [beta estimate: 0.51, 95% CI: (−0.46–1.50), *P* = 0.30] between [^18^F]-MK-6240 SUVR levels in Braak Stages I and II regions and age were observed with annual changes in the memory composite *Z*-score.

Figure 3 illustrates memory composite *Z*-score slopes over the range of baseline Braak Stages I–II [^18^F]-MK-6240 SUVR levels present in each age group based on the mean (< 70 years and > 70 years).

The background (points) shows the individual observed memory composite *Z*-scores. No statistically significant interaction between [^18^F]-MK-6240 SUVR levels in Braak Stages I and II regions and age were observed with annual changes in the memory composite *Z*-score [beta estimate: 0.51, 95% CI: (−0.46–1.50), *P* = 0.30].

### PET biomarkers and sex

**Figure 4:**
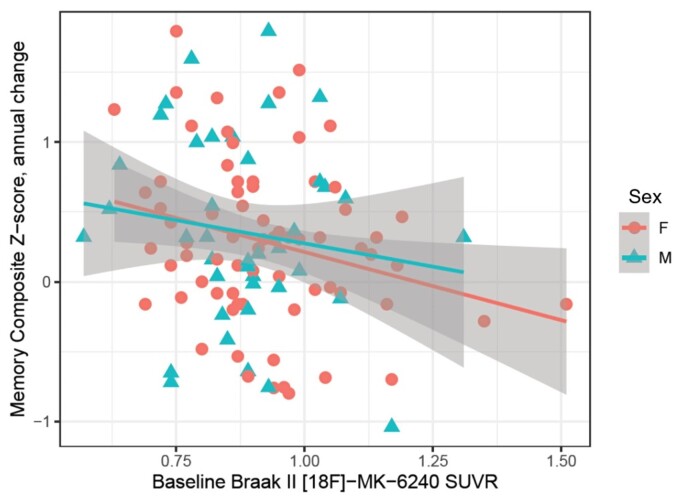
**Association between baseline Braak Stages I–II [^18^F]-MK-6240 SUVR and longitudinal memory decline, stratified by sex.** In cognitively unimpaired participants, no statistically significant interaction (beta estimate: 0.16, 95% CI: (−1.13–1.44), *P* = 0.81) between [^18^F]-MK-6240 SUVR levels in Braak Stages I and II regions and sex were observed with annual changes in the memory composite *Z*-score.

No statistically significant interaction between [^18^F]-MK-6240 SUVR levels in Braak Stages I and II regions and sex were observed with annual changes in the memory composite *Z*-score (beta estimate: 0.16, 95% CI: (−1.13 to 1.44), *P* = 0.81) (Fig. 4).

## Discussion

This prospective cohort study examined the prognostic accuracy of tau-PET in predicting cognitive trajectories between groups stratified on tau neurofibrillary tangles in the medial temporal lobe {[^18^F]-MK-6420} and neocortical Aβ plaques {[^18^F]-AZD-4694} in relatively healthy, initially CU elderly individuals. The majority of subjects, 70% (*n* = 78), were categorized as A−T−, 8% (*n* = 9) as A−T+, 12% (*n* = 13) as A + T− and the remaining 10% (*n* = 11) as A + T + . A similar distribution was observed in a longitudinal observational study conducted by Betthauser *et al*.^6^, who also employed [^18^F]-MK-6240 and focused on medial temporal tau, where 74% (*n* = 124) were classified as A−T−, 3% (*n* = 5) as A−T+, 14% (*n* = 23) as A + T− and 9% (*n* = 15) as A + T + .^20^ While the proportion of A−T + individuals in our study is slightly higher than other recent *in vivo* PET studies, our findings may be attributable to the fact that tau abnormalities were defined based on medial temporal brain regions, which are considered to occur earlier than widespread neocortical tau abnormalities.^4,21^ In neuropathological studies, tau abnormalities in early Braak regions in the absence of Aβ deposition are referred to as primary age-related tauopathy.^22^

Our primary analysis showed a significant effect of Braak Stages I–II [^18^F]-MK-6420 SUVR on change in the annual memory composite *Z*-score at baseline and longitudinally, independently of age, sex and neocortical Aβ-PET SUVR. Participants with pathological Aβ and medial temporal tau (A + T+) exhibited moderate cognitive decline over the follow-up period. No significant change was observed for the A−T+, A + T− and A−T− groups during the follow-up period, though it should be noted that the A−T + group was small. These results indicate that the accumulation of Aβ plaques and neurofibrillary tangles—detectable with PET—in early neurofibrillary tangle stage regions leads to abnormal cognitive decline during the preclinical stage of Alzheimer’s disease.^23^ This supports the use of PET biomarker stratification to define the construct of preclinical Alzheimer’s disease and insinuates that the associated memory decline within this presymptomatic stage may begin earlier than previously suggested.^24^ This also signifies that tau-PET can act as a powerful prognostic marker that can accurately predict rates of future cognitive decline, several years prior to clinical impairment. As such, our findings have implications for studies that examine the associations between Aβ and cognitive performance where assessments of tau pathology are not included. Levels of Aβ are poorly associated with the severity of dementia in Alzheimer’s disease individuals, while tau neurofibrillary tangles levels are more closely associated with cognitive symptoms.^10^ Given this information, the ability to measure tau burden in living persons is crucial in establishing an accurate diagnosis of Alzheimer’s disease and predicting one’s risk towards clinical progression.^25^

Our results indicate that tau-PET may serve as a robust biomarker for the identification of Aβ-positive patients with tau-positivity (A + T+).^26^ Since these individuals have a greater susceptibility to experiencing future cognitive decline, using this population for clinical trial designs would be beneficial for studying those at risk for Alzheimer’s disease.^27^ Some important derivations could be made based on our findings. First, incorporating tau-PET abnormalities as an inclusion criterion for recruitment in clinical trials would provide greater insight into the spatial topography of tauopathy and serve as a useful predictive model of cognitive decline. Some arguments could be made for the additional inclusion of tau pathology for Aβ monotherapy trials since it would help with obtaining a more targeted population.^28^ Second, it is highly feasible when designing clinical trials to stratify participants based on the presence of early tau. Third, a longer duration of trials may be required during the preclinical or prodromal Alzheimer’s disease stages to better observe differences in cognitive decline, as measured differences within one year may be too small. Fourth, tau aggregation outside the medial temporal lobe is also associated with more severe cognitive decline. Recent findings from our group demonstrate that the presence of tau in early Braak regions may have high sensitivity for capturing the risk of cognitive decline, whereas the accumulation of tau in more advanced Braak regions is associated with greater memory dysfunction.^16^ Thus, clinical trials focusing on the symptomatic phases of Alzheimer’s disease may wish to stratify subjects based on the presence of tau-PET abnormalities in more advanced Braak stages. Further neuroimaging research is merited to answer this particular question. Overall, our findings contribute to the body of literature that demonstrates the utility of tau-PET as an accurate predictor of cognitive trajectories for patients with preclinical Alzheimer’s disease.

This study adds to the overall body of recent neuroimaging reports assessing Alzheimer’s disease biomarkers in CU elderly.^6,7,9,11,29,30^ It also supports recent findings that demonstrate tau-PET as an accurate prognostic marker in preclinical and prodromal Alzheimer’s disease.^10^ However, compared with these longitudinal studies, our study used the [^18^F]-MK-6420 PET tracer, focusing on imaging the medial temporal regions for the detection of very early tau in CU individuals. Moreover, earlier work has highlighted that the topography of tau-PET uptake is associated with domain-specific cognitive decline.^31^ More recent work has also provided evidence for tau-PET association with executive dysfunction in CU individuals.^7^ Thus, it should be noted that tau-PET can be used to capture cognitive decline in different domains as well.

Future work should include a larger sample size, a longer study period and be paired with other tests of other cognitive domains to assess whether there will be an improvement in the accuracy of tau-PET as a prognostic marker for preclinical Alzheimer’s disease.

Presently, both Aβ plaques and tau neurofibrillary tangles can be detected *in vivo* using PET imaging techniques, allowing for the spatiotemporal characterization of these biomarkers during one’s lifetime.^6,7^ With Aβ tracers—^18^F-florbetapir, ^18^F-flutemetamol and ^18^F-florbetaben—and the first tau tracer, ^18^F-flortaucipir, recently approved by the FDA, Alzheimer’s disease molecular imaging is transitioning from a preclinical modality towards a fully clinically useful tool.^5^ This new technique can improve the diagnostic accuracy of our present clinical criteria when assessing individuals with cognitive impairment, which currently has limited sensitivity and specificity relative to autopsy.^5^

The strengths of this study include its longitudinal cohort design, use of multimodal imaging (Aβ-PET and tau-PET) and availability of longitudinal logical memory and RAVLT scores. However, some methodological limitations must be considered when interpreting this study. The first is that the memory tests used were written in French, which may not be well validated for non-English participants. Moreover, it is important to note that the Translational Biomarkers in Ageing and Dementia cohort is a convenience sample of individuals who are motivated to participate in a study about ageing and dementia, therefore introducing recruitment and sampling biases. Another shortcoming is the limited post-mortem data available for the [^18^F]-MK-6420 tau-PET tracer.^14^ A greater availability of autopsy studies would help with our understanding of the extent to which [^18^F]-MK-6240 binds to tau neurofibrillary tangle levels. Additionally, the memory tests used in this study are standard. There are other tests that may be more optimally positioned and sensitive to detect very early cognitive changes in CU individuals. It is also important to emphasize that tau abnormality in the present study was defined based on medial temporal brain regions, which may partially account for the higher proportion of A-T + reported in our study, as compared with other recent PET imaging studies.^4^ Future studies should address whether APOEɛ4 genotype independently predicts prospective cognitive decline beyond Aβ-PET and tau-PET levels, given the association between APOEɛ4 genotype and both Aβ-PET and medial temporal tau-PET. Furthermore, our study did not investigate changes in diagnosis (i.e. CU to mild cognitive impairment) and it was correspondingly not possible to determine hazard ratios for clinical progression. Therefore, larger studies with longer follow-up durations are warranted.

## Conclusion

In this prognostic study, the [^18^F]-MK-6240 tau-PET tracer showed strong prognostic utility as an accurate predictor of longitudinal cognitive change, especially in cognitively unimpaired individuals during the preclinical stage of Alzheimer’s disease.

## Data Availability

McGill University has succinctly reviewed all requests pertaining to raw and analysed data and all other related material to ensure and verify that the intellectual property and confidentiality statements abide by ethical standards. If and when anonymized data is requested, only a qualified academic investigator may share the information with the senior author of the study. This agreement will be permitted for the sole purpose of the replication procedures and results presented in this paper. A material transfer agreement will therefore be required for the sharing and release of relevant data and materials. Otherwise, data will not be publicly available due to the compromised privacy and confidentiality of research participants.

## Abbreviations

Aβ =: amyloid-β
AD =: Alzheimer’s disease
ADNI =: Alzheimer’s disease neuroimaging initiative
CU =: cognitively unimpaired
PART =: primary age-related tauopathy
RAVLT =: Rey auditory verbal learning test
SUVR =: standardized uptake value ratio
TRIAD =: translational biomarkers in ageing and dementia
WMH =: white matter hyperintensity

## References

[1] Knopman DSK, Arisi JEP, Alviati AS, et al Neuropathology of cognitively normal elderly. J Neuropathol Exp Neurol. 2003;62(11):1087–1095. 14656067 10.1093/jnen/62.11.1087

[2] Schmitt FA, Davis DG, Wekstein DR, Smith CD, Ashford JW, Markesbery WR. “Preclinical” AD revisited: Neuropathology of cognitively normal older adults. Neurology. 2000;55(3):370–376. 10932270 10.1212/wnl.55.3.370

[3] Jack CR, Bennett DA, Blennow K, et al NIA-AA Research framework: Toward a biological definition of Alzheimer’s disease. Alzheimers Dement. 2018;14(4):535–562. 29653606 10.1016/j.jalz.2018.02.018 PMC5958625

[4] Jack CR, Wiste HJ, Therneau TM, et al Associations of amyloid, tau, and neurodegeneration biomarker profiles with rates of memory decline among individuals without dementia. JAMA. 2019;321(23):2316–2325. 31211344 10.1001/jama.2019.7437 PMC6582267

[5] Rabinovici GD, Gatsonis C, Apgar C, et al Association of amyloid positron emission tomography with subsequent change in clinical management among medicare beneficiaries with mild cognitive impairment or dementia. JAMA. 2019;321(13):1286–1294. 30938796 10.1001/jama.2019.2000 PMC6450276

[6] Betthauser TJ, Koscik RL, Jonaitis EM, et al Amyloid and tau imaging biomarkers explain cognitive decline from late middle-age. Brain. 2020;143(1):320–335. 31886494 10.1093/brain/awz378 PMC6935717

[7] Sperling RA, Mormino EC, Schultz AP, et al The impact of amyloid-beta and tau on prospective cognitive decline in older individuals. Ann Neurol. 2019;85(2):181–193. 30549303 10.1002/ana.25395 PMC6402593

[8] Therriault J, Benedet AL, Pascoal TA, et al Association of plasma P-tau181 with memory decline in non-demented adults. Brain Commun. 2021;3(3):fcab136. 10.1093/braincomms/fcab136 PMC8249102 34222875

[9] Lowe VJ, Bruinsma TJ, Wiste HJ, et al Cross-sectional associations of tau-PET signal with cognition in cognitively unimpaired adults. Neurology. 2019;93(1):E29–E39. 31147421 10.1212/WNL.0000000000007728 PMC6659005

[10] Ossenkoppele R, Smith R, Mattsson-Carlgren N, et al Accuracy of tau positron emission tomography as a prognostic marker in preclinical and prodromal Alzheimer disease: A head-to-head comparison against amyloid positron emission tomography and magnetic resonance imaging. JAMA Neurol. 2021;78(8):961–971. 34180956 10.1001/jamaneurol.2021.1858 PMC8240013

[11] Schöll M, Lockhart SN, Schonhaut DR, et al PET imaging of tau deposition in the aging human brain. Neuron. 2016;89(5):971–982. 26938442 10.1016/j.neuron.2016.01.028 PMC4779187

[12] Fleisher AS, Pontecorvo MJ, Devous MD, et al Positron emission tomography imaging with [18F]-flortaucipir and postmortem assessment of Alzheimer disease neuropathologic changes. JAMA Neurol. 2020;77(7):829–839. 32338734 10.1001/jamaneurol.2020.0528 PMC7186920

[13] Lowe VJ, Lundt ES, Albertson SM, et al Tau-positron emission tomography correlates with neuropathology findings. Alzheimers Dement. 2020;16(3):561–571. 31784374 10.1016/j.jalz.2019.09.079 PMC7067654

[14] Pascoal TA, Shin M, Kang MS, et al In vivo quantification of neurofibrillary tangles with [18F]-MK-6240. Alzheimers Res Ther. 2018;10(1):74. 30064520 10.1186/s13195-018-0402-y PMC6069775

[15] Therriault J, Benedet AL, Pascoal TA, et al Association of apolipoprotein e ɛ4 with medial temporal tau independent of amyloid-β. JAMA Neurol. 2020;77(4):470–479. 31860000 10.1001/jamaneurol.2019.4421 PMC6990684

[16] Therriault J, Pascoal TA, Lussier FZ, et al Biomarker modeling of Alzheimer’s disease using PET-based braak staging. Nat Aging. 2022;2:526–535. 10.1038/s43587-022-00204-0 PMC10154209 37118445

[17] Jack CR, Wiste HJ, Weigand SD, et al Defining imaging biomarker cut points for brain aging and Alzheimer’s disease. Alzheimers Dement. 2017;13(3):205–216. 27697430 10.1016/j.jalz.2016.08.005 PMC5344738

[18] Mielke MM, Frank RD, Dage JL, et al Comparison of plasma phosphorylated tau species with amyloid and tau positron emission tomography, neurodegeneration, vascular pathology, and cognitive outcomes. JAMA Neurol. 2021;78(9):1108–1117. 34309632 10.1001/jamaneurol.2021.2293 PMC8314178

[19] Pascoal TA, Therriault J, Benedet AL, et al 18F-MK-6240 PET for early and late detection of neurofibrillary tangles. Brain. 2020;143(9):2818–2830. 32671408 10.1093/brain/awaa180

[20] Braak H, Braak E, Braak E. Staging of Alzheimer’s disease-related neurofibrillary changes. Frankfurt: Goethe University; 1995. 10.1016/0197-4580(95)00021-6 7566337

[21] Crary JF, Trojanowski JQ, Schneider JA, et al Primary age-related tauopathy (PART): A common pathology associated with human aging. Acta Neuropathol. 2014;128(6):755–766. 25348064 10.1007/s00401-014-1349-0 PMC4257842

[22] Laslett P . The Third Age and the Disappearance of Old Age. New York, NY: Acta Neuropathologica; 2014.

[23] Jack CR, Knopman DS, Jagust WJ, et al Tracking pathophysiological processes in Alzheimer’s disease: An updated hypothetical model of dynamic biomarkers. Lancet Neurol. 2013;12(2):207–216. 23332364 10.1016/S1474-4422(12)70291-0 PMC3622225

[24] Lu M, Pontecorvo MJ, Devous MD, et al Aggregated tau measured by visual interpretation of flortaucipir positron emission tomography and the associated risk of clinical progression of mild cognitive impairment and Alzheimer disease: Results from 2 phase III clinical trials. JAMA Neurol. 2021;78(4):445–453. 33587110 10.1001/jamaneurol.2020.5505 PMC7885097

[25] Strikwerda-Brown C, Hobbs DA, Gonneaud J, et al Association of elevated amyloid and tau positron emission tomography signal with near-term development of Alzheimer disease symptoms in older adults without cognitive impairment. JAMA Neurol. 2022;79(10):975–985. 35907254 10.1001/jamaneurol.2022.2379 PMC9339146

[26] Hanseeuw BJ, Betensky RA, Jacobs HIL, et al Association of amyloid and tau with cognition in preclinical Alzheimer disease: A longitudinal study. JAMA Neurol. 2019;76(8):915–924. 31157827 10.1001/jamaneurol.2019.1424 PMC6547132

[27] Kwan ATH, Arfaie S, Therriault J, Rosa-Neto P, Gauthier S. Lessons learnt from the second generation of anti-amyloid monoclonal antibodies clinical trials. Dement Geriatr Cogn Disord. 2021;49(4):334–348. 10.1159/000511506 33321511

[28] Aschenbrenner AJ, Gordon BA, Benzinger TLS, Morris JC, Hassenstab JJ. Influence of tau PET, amyloid PET, and hippocampal volume on cognition in Alzheimer disease. Neurology. 2018;91(9):e859–e866. 30068637 10.1212/WNL.0000000000006075 PMC6133625

[29] Knopman DS, Petersen RC, Jack CR. A brief history of “Alzheimer disease”: Multiple meanings separated by a common name. Neurology. 2019;92(22):1053–1059. 31028129 10.1212/WNL.0000000000007583 PMC6556090

[30] Ossenkoppele R, Schonhaut DR, Scholl M, et al Tau PET patterns mirror clinical and neuroanatomical variability in Alzheimer’s disease. Brain. 2016;139(5):1321–1324. 26962052 10.1093/brain/aww027 PMC5006248

[31] James ML, Gambhir SS. A molecular imaging primer: Modalities, imaging agents, and applications. Physiol Rev. 2012;92(2):897–965. 22535898 10.1152/physrev.00049.2010

